# The Dentist’s Burden

**Published:** 1899-06

**Authors:** F. S. Caspar

**Affiliations:** Austin, Texas


### The Dentist's Burden.

BY F. S. CASPAR, D. D. S., AUSTIN, TEXAS.

When we consider that a patient who comes into our office to place himself, or herself, as the case may be, under our care for treatment, is in reality doing us the honor to show preference over other practitioners, we owe to them not only our best and most skillful attention, but we should exercise the greatest care that in serving them we do not add to their affliction by communicating disease germs from other patients.

Our first consideration should be to see that our instruments are cleansed and sterilized; fresh antiseptic napkins should be supplied; the hands, and especially the finger nails, should be thoroughly cleansed, and above all things we should see that the spittoon is pure and fresh, free from obnoxious odors.

In this connection, let us say, we are happy to state that certain manufacturers of dental goods have succeeded in placing on the market an improved apparatus for sterilizing instruments and other things used about the operating chair, which, for simplicity and cheapness, cannot be excelled. And an improved fountain spittoon also, which seems to be a model of perfection, ornamental in design, self-cleansing and cheap. No dentist can well afford to deny himself these necessary additions to a well kept office.

### SOAP AS A DENTIFRICE (?).

In rummaging through a lot of old journals, our attention was attracted to an article in the *Dental Headlight,* January number, 1890, under the head of "Care of the Teeth," which reads as follows: "At the meeting in Berlin last spring, of the German Association of American Dentists, the best means of preserving the teeth was discussed, and Dr. Richter of Breslau, said: 'We know that the whole method of correctly caring for the teeth can be expressed in two words—"brush, soap." In these two things we have all that is needful for the preservation of the teeth. The preparations not containing soap are not to be recommended, and if they contain soap all other ingredients are useless, except for the purpose of making their taste agreeable.' " This article was originally taken from the *Scientific American.*

We can remember when once upon a time we recommended the use of Castile soap as the proper dentifrice, but latterly, we have been convinced of our error. We quote from *The Dental Review*, December 15, 1896, page 927, Odontographic Society of Chicago, Dr. E. L. Clifford: "Dr. McWhinney spoke in his paper about the bacteria of pus, and I would like to ask the members of this Society to look in the mouths of their patients for the *chromogenic bacteria* that are found around the gingival margins of the teeth, spoken of by many dentists as the 'brick dust' or 'red' variety. I would like to have the members of this Society for a year count the number of patients they find with the red variety of chromogenic bacteria on the margins of the teeth, and I would like to have them ask these questions: whether they are in the habit of using, or have used in the past, *any kind* of soap as a dentifrice?"

Dr. E. L. Clifford has made a study of bacteriology, and his opinion is based upon established facts. The conclusion is, therefore, that soap dentifrices are not to be recommended.

